# Non-Contact Damage Detection under Operational Conditions with Multipoint Laservibrometry

**DOI:** 10.3390/s20030732

**Published:** 2020-01-28

**Authors:** Xiaodong Cao, Christian Rembe

**Affiliations:** Institute of Electrical Information Technology, Clausthal University of Technology, 38678 Clausthal-Zellerfeld, Germany; rembe@iei.tu-clausthal.de

**Keywords:** damage detection, multipoint laser-Doppler vibrometer, structural health monitoring, operational condition, operational deflection shape, laser ablation, non-destructive testing, laser–Doppler vibrometry

## Abstract

Scanning laser–Doppler vibrometry (SLDV) can localize and visualize damages in mechanical structures. In order to enable scanning, it is necessary to repeat the vibration. Therefore, this technique is not suited to detect emerging hazards in working machinery that change the vibration behavior. A common technique for such cases is monitoring the vibration excited by machine operation with accelerometers. This technique requires mechanical coupling between sensors and the measurement object, which influences the high-frequency vibration responses. However, in the low-frequency range, local damages do not shift resonances or distort operational deflection shapes (ODS) significantly. These alterations in the vibration behavior are tiny and hard to detect. This paper shows that multipoint laservibrometry (MPV) with laser excitation can measure these effects efficiently, and it further demonstrates that damages influence ODSs at frequencies above 20 kHz much stronger than at frequencies below 20 kHz. In addition, ODS-based damage indices are discussed; these are highly sensitive to minute visible changes of the ODSs. In order to enhance the sensitivity of hazard detection, the response vector assurance criterion value is computed and evaluated during operation. The capabilities and limitations of the methodology on the example of a cantilever with manually emerging damage are demonstrated.

## 1. Introduction

Structural damages in mechanical systems triggered by material fatigue have an essential influence on live time and performance. Different non-destructive methods are widely applied for damage detection, such as ultra-sound, X-rays, or eddy current. These methods are used to detect damages during maintenance. Vibration-based damage detection techniques can be applied directly to working machinery; therefore, these techniques are attractive for structural health-monitoring (SHM) [1,2,3]. Damage occurrence changes physical dynamic properties (like mass, damping, and stiffness) of a structure. These kinds of changes are visible in the form of natural frequency shifts and influence the associated operational deflection shapes (ODS) or mode shapes. Thus, vibration-detection techniques can provide means to detect damages.

In the last few decades, vibration-based damage detection techniques under laboratory conditions have been extensively studied. The influence from the environment is in such cases negligible and the structure is reproducibly excited, for example, by a modal shaker with a predefined signal. Damage detection under normal machinery operational conditions is still a challenge for research because influences from the operation and the environment are generally not negligible and lead to changes in dynamic properties of the structure as well. Investigations show that post-processing methods based on singular value decomposition (SVD) increase the accuracy and the robustness against measurement uncertainties [4,5,6] of the damage detection results. However, the vibration excited by working machinery is much more complicated.

Furthermore, damage discovery in the very early stage of a local damage occurrence is still an aspired goal of all the detection techniques, especially for SHM. Commonly, the ODSs are measured simultaneously by acceleration sensor arrays. The useful frequency range of an acceleration sensor is limited by its mounted resonance frequency [7]. The typical frequency range of most commercial (piezo) acceleration sensors used for vibration testing is at a few 10 kHz [8]. In the low-frequency range, the environmental or operational influences, such as those from a rotating machine, are relatively strong. This can make damage detection difficult. In the frequency region above 20 kHz, the amplitude of machine operation is normally much smaller than in the region below 20 kHz [9]. In addition, changes of dynamic properties due to local damages are much more sensitive in frequency regions with higher order ODSs, which are often above 20 kHz. Thus, wide frequency-range measurements with information over 20 kHz can increase sensitivity of the detection. In the high-frequency range, the ultrasonic field may be disturbed due to the mechanical coupling between the acceleration sensor and the test object. The vibration response measured by an acceleration sensor is generally dependent on the efficiency of the mechanical coupling [7,10]. Variations in the coupling conditions may cause significant degrees of uncertainty to the ODS measurements by acceleration sensor arrays in the high frequency range. This limitation can be avoided by non-contact measurement techniques. Scanning laser-Doppler vibrometry (SLDV) has been introduced to local damage detection in the last few decades [11]. A laser-Doppler vibrometrer (LDV) has a flat frequency response in a frequency range, only limited by the electronics and data processing. Frequency ranges are even possible up to the MHz range [12,13,14,15]. Research results show that small local damages can be effectively detected by SLDV especially at the frequency region above 20 kHz [16,17]. However, the SLDV is not applicable for damage detection under operation because the vibration has to be repeated to enable scanning. The vibration from working machinery is generally not repeatable.

This paper shows an optimized non-contact measurement methodology for damage detection under normal machinery operational conditions. ODSs are measured by a multipoint vibrometer (MPV) [18,19,20]. The MPV allows simultaneous vibration measurements with 48 channels. In order to increase the sensitivity of the detection, all the resonances of the structure in the entire measurement frequency range should be taken into account. The resonances are commonly not all sufficiently excited by normal machine operation. Therefore, a nanosecond laser is utilized to introduce very broadband vibration excitations (i.e., significantly over 100 kHz) by laser ablation [21,22,23,24], additionally to the operational excitation. The frequencies of resonances are estimated by using the frequency domain decomposition method, which is successfully deployed in operational modal analysis [25]. It estimates the corresponding dominant modes at a frequency by evaluating singular value decompositions (SVD) of the spectral density matrix. The spectral density matrix is received from the vibration response data. It is important to use such output only methods because it is quite difficult to measure the actual excitation mechanisms in working machinery accurately. The ODSs of the intact structure are then compared to that of the damaged structure, at the resonance frequencies in the entire measurement frequency range. To compare the ODSs, we use the response vector assurance criterion (RVAC) to measure the correlation between the ODS pairs [26]. By taking the mean value from RVAC values over all selected frequency points, the relative damage quantification indicator (DRQ) can be calculated to describe the damage state with a single value [27].

We demonstrate the capabilities and limitations of our methodology on an example of a cantilever beam in three damage levels. The damage levels are introduced by varying the depth of a blind hole on the cantilever. In order to simulate the operational condition, a small metal wheel driven by an electric motor grinds the cantilever slightly. The results show that all three damage levels are detectable under operational condition directly. Finally, we compare the results from our method with the detection result from a constructed SLDV measurement to illustrate the advantage of our methodology.

## 2. Methods

### 2.1. Damage Index

A damaged structure has a different dynamic behavior and therefore a different ODS compared to its intact state. There is a simple and widely used way to check if the structure is damaged. Namely, to calculate the correlation between the ODSs of the structure under two different states: the potentially damaged state and its intact state. If the structure is undamaged, the two ODSs will be correlated and the correlation is then one. The degree of correlation decreases if the damage grows. The correlation at every frequency point ω with RVAC can be calculated by [26]

(1) RVAC(ω)=‖∑i=1Nhdi(ω)hiH(ω)‖2∑i=1N[hdi(ω)hdiH(ω)]∑i=1N[hi(ω)hiH(ω)]=‖hH(ω)hd(ω)‖2(hH(ω)h(ω))(hdH(ω)hd(ω)),

with

(2) h(ω)=[h1(ω)h2(ω)⋮hN(ω)] and hd(ω)=[hd1(ω)hd2(ω)⋮hdN(ω)].

h(ω) is the response vector of the intact or reference structure, its element $h_{i}$ is the response at the degree of freedom *i*, such as a measurement point in one measurement direction. *N* is the number of degree of freedoms, and hd(ω) is the response vector of the damaged structure, respectively. The index superscript d on the left stands for damaged state. The index superscript H means Hermitian transpose, and ‖·‖ stands for Euclidean norm. The response vectors can be calculated values, such as frequency response functions from a finite element model, or directly measured values, such as velocities—for example measured by an LDV. If the modal vector instead of the response vector is used at the corresponding resonance frequency point, then the RVAC value will be the well-known modal assurance criterion [28]. This criterion is usually applied to find the corresponding mode shapes between an experiment and a finite element model by finite element model updating problems [29,30,31,32]. For damage detection, it is practical to use one scalar value to describe the damage state of the structure. In order to achieve this request, the mean value of the RVAC can simply be used. This is defined as the detection and relative damage quantification indicator (DRQ) [26,27]:

(3) $$\mathrm{DRQ}=\frac{1}{N_{\omega }}\sum_{i=1}^{N_{\omega }}\mathrm{RVAC}\left(\omega_{i}\right),$$

where $N_{\omega }$ is the number of used frequencies $\omega_{i}$.

The ODSs measured under operational conditions could be noisy. One possibility to increase the noise robustness of ODS-based damage detection is to apply SVD-based methods. We chose the frequency domain decomposition method, which was developed to estimate modal parameters in operational modal analysis. It calculates the dominate modes at a frequency point by applying SVD on the spectral density matrix $G_{h}\left(\omega \right)$ of the measured time response vector h(t) [33]

(4) $$G_{h}\left(\omega \right)=U\;S\;U^{H}\;\mathrm{with}\;U=\left[\begin{array}{cc} \begin{array}{cc} u_{1}\left(\omega \right) & u_{2}\left(\omega \right) \end{array} & \begin{array}{cc} \cdots & u_{n}\left(\omega \right) \end{array} \end{array}\right],$$

where S is a diagonal matrix with the singular values (diagonal elements) $s_{1}^{2}\left(\omega \right)>s_{2}^{2}\left(\omega \right)>\cdots >s_{n}^{2}\left(\omega \right)$ sorted in descending order. The corresponding singular vectors $u_{1}\left(\omega \right)$ to $u_{n}\left(\omega \right)$ can be interpreted as the sub-components of the ODS at the corresponding frequency point ω. Their participations in the ODS are sorted by their corresponding singular values $s_{1}^{2}\left(\omega \right)$ to $s_{n}^{2}\left(\omega \right)$. This means that $u_{1}\left(\omega \right)$ is the most dominant sub-component of the ODS at frequency ω because its corresponding singular value $s_{1}^{2}\left(\omega \right)$ is the biggest singular value [5]. The singular values $s_{1}^{2}\left(\omega \right)$ can be plotted over the frequency, i.e., the singular value spectrum. Every peak in this spectrum indicates a possible resonance at this frequency region. The signal-to-noise ratio (SNR) of measured ODSs in this frequency region is much better than the region between two adjacent resonances, especially in the anti-resonance region. Therefore, we use the peaks in the singular value spectrum of $s_{1}^{2}\left(\omega \right)$ to define the frequency points $\omega_{i}$ in Equation (3), and take $u_{1}\left(\omega \right)$ from the intact structure and ud1(ω) from the damaged structure, instead of h(ω) and hd(ω) in Equation (1) for each $\omega_{i}$ to calculate the DRQ. Consequently, it is more robust against noises than the DRQ calculated directly by measured ODSs. The main steps of the DRQ calculation are shown in the following:

Step1: Measure the velocities at the measurement points on the intact and damaged structure by the MPV. These are the time response vectors h(t) and hd(t).
Step 2: Calculate the spectral density matrices Gh(ω) and Gdh(ω) of the time response vectors h(t) and hd(t). Use Equation (4) to calculate the singular value spectrum of the intact structure $s_{1}^{2}\left(\omega \right)$, the damaged structure sd12(ω), and their corresponding singular vectors $u_{1}\left(\omega \right)$ and ud1(ω).
Step 3: Find the peaks in the singular value spectrum $s_{1}^{2}\left(\omega \right)$ and their corresponding frequency points $\omega_{i}$. Then, calculate the RVAC values at each frequency point $\omega_{i}$ by using $u_{1}\left(\omega \right)$ and ud1(ω) instead of h(ω) and hd(ω) in Equation (1):
  (5) RVAC(ωi)=‖u1H(ωi)ud1(ωi)‖2(u1H(ωi)u1(ωi))(ud1H(ωi)ud1(ωi)).
Step 4: Calculate the DRQ value by using Equation (3).

### 2.2. Measurement Setup

**Figure 1:**
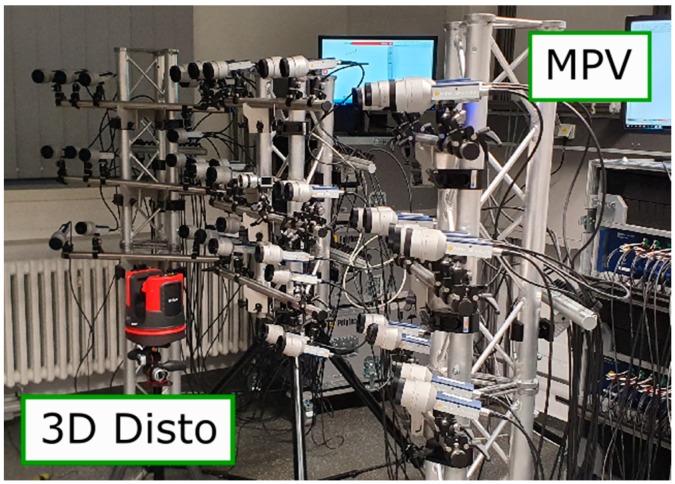
The MPV-800 and the 3D Disto.

The accuracy of the damage detection results depends largely on the quality of the measured ODSs. In case the vibration is not repeatable, which is generally true for the SHM, the ODS has to be simultaneously measured. High sensitivity to local damage requires high spatial resolution and wide frequency range. These two goals are very difficult to achieve at the same time because both of them increase requirements for data acquisition performance drastically. Therefore, compromises have to be made. We use our MPV system MPV-800 (Polytec GmbH, Waldbronn, Germany, Figure 1) to measure ODSs [34]. This system is capable of performing up to 48 measurements at the same time. Every measurement channel has a bandwidth from 0 to 100 kHz. In order to obtain 3D vibration information at one measurement point, measurements by three laser beams (i.e., three channels of MPV) from three different directions are measured. The 3D vibration measurements of the MPV and the SLDV work on the same principle. The 3D velocity vector is calculated by a coordinate transformation [35,36]. The transformation matrix contains Euler angles of the directions of the laser beams. MPV-800 employs 3D Disto (Leica Geosystems GmbH, Munich, Germany) to measure the Euler angles (Figure 1) [37].

To increase the detection sensitivity, the full detection frequency range of the MPV should be exploited, i.e., from 0 to 100 kHz. Therefore, we excite the structure externally with extremely short laser pulses. We utilize a neodymium-doped yttrium aluminum garnet (Nd:YAG) laser (Quantel Brilliant EaZy, Lumibird Group, Les Ulis Cedex, France) with a wavelength of 1064 nm, pulse duration of 5 ns, and pulse energy of 300 mJ. The Nd:YAG laser provides different modes of laser emission: the single shot mode and repetitive shot mode with variable repetition rate (up to 10 Hz). In repetitive shot mode, the Nd:YAG laser emits light pulses spaced in time due to a pre-set repetition rate. The pulse width and peak power do not change for different repetition frequencies. Higher repetition rate allows more averaging. Therefore, we operated the Nd:YAG laser at the maximum repetition rate of 10 Hz. The Nd:YAG laser provides a transistor-transistor logic (TTL) signal, which is synchronized to the laser pulses. After starting the measurement in the MPV-software, the MPV measurement is synchronized to the next TTL signal (which corresponds to the start of the next laser pulse). The Nd:YAG laser excitation is suitable for investigations of plate-like structures on running test benches. For large structures, laser pulses with much more energy may be required [23]. Alternatively, piezoelectric shakers or lead zirconate titanate (PZT) patches can be used to achieve high frequency excitation [16] if the test environment does not allow laser excitation. The excitation bandwidth may be not as wide as by laser excitation. However, it is still possible to achieve an acceptable broad excitation bandwidth by applying multiple piezoelectric shakers or PZTs with different excitation frequency ranges.

### 2.3. Experiment

**Figure 2:**
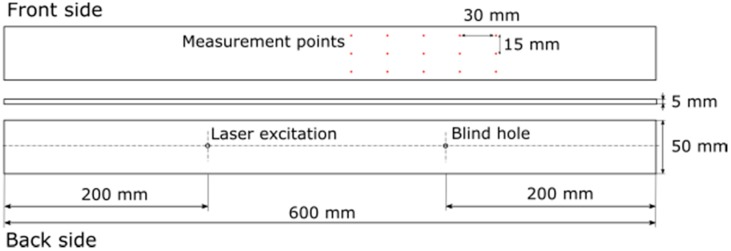
Cantilever beam.

An aluminum cantilever beam is studied to prove the capability of our method. A blind hole is drilled on the back side of the cantilever beam (Figure 2). We define three damage levels with different depths of the blind hole for this experiment. The data on the blind hole at three damage levels are listed in Table 1.

**Figure 3:**
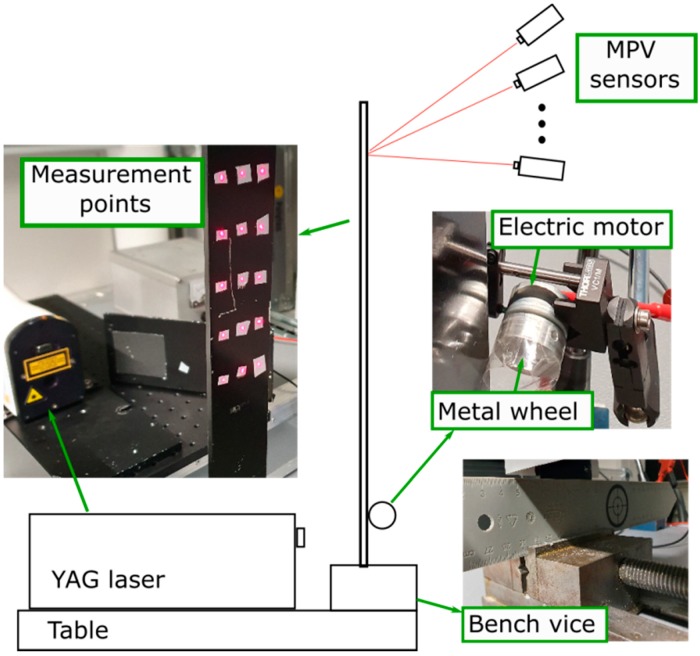
Measurement setup.

Figure 3 shows the measurement setup schematically. Fifteen measurement points are defined on the cantilever beam. All measurement points are marked by reflection stickers as shown in Figure 3. The cantilever beam is fixed by a bench vice. To demonstrate the existence of machine operational conditions, we ground the cantilever lightly by a small metal wheel. An electric motor drove the wheel at about 2000 rpm. The surface of the metal wheel is covered with a piece of scotch tape to avoid direct friction between these two metal surfaces because such friction is not common in working machinery.

## 3. Results

Theoretically, the ODSs from two measurements should be the same if no damage occurs on the structure during these measurements. In addition, the RVAC values should be all equal to one. However, it is not true in practice. Mechanisms under operational conditions and the measurement uncertainties probably influence the RVAC values. Therefore, we need to find a way to define a reliable DRQ value as a reference value for the intact structure. It should be only sensitive to damages in the structure. Therefore, we captured five measurements on the intact cantilever beam and calculated the RVAC values between them first. All measurements were sampled with 250 kHz for 4 s. Then, we drilled the blind hole in it. As mentioned in Section 2.1, only measured data in the frequency spectrum around the resonance points are used. These data have a good SNR and will be used to calculate the RVAC values. In this paper, we pick all the peaks in the singular value spectrum of the measurement 1, and define a narrow band around each peak. These narrow bands are then used to find the corresponding resonance frequencies of further measurements by picking the peaks in their singular value spectrums.

**Figure 4:**
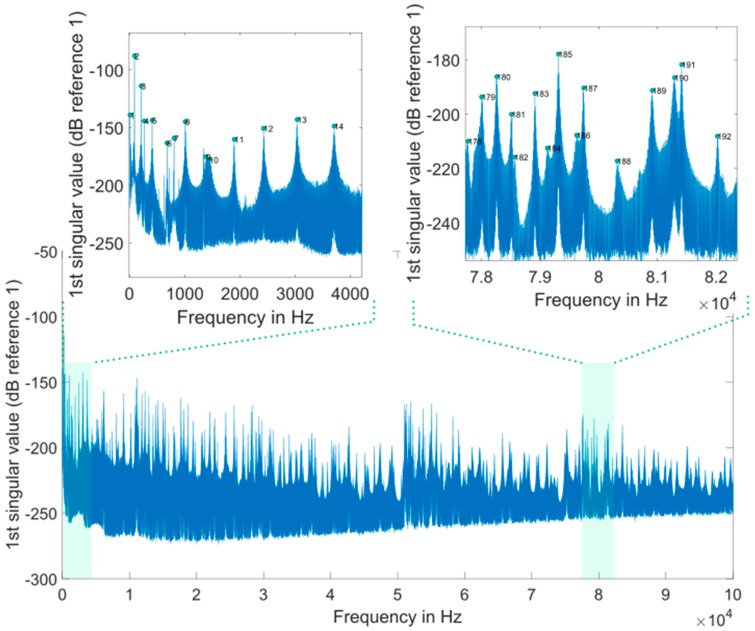
First singular value spectrum of measurement 1.

**Figure 5:**
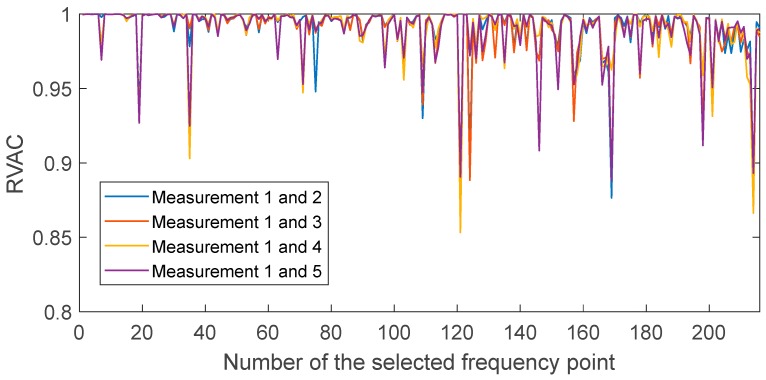
RVAC curves calculated from measurements 1 to 5.

**Figure 6:**
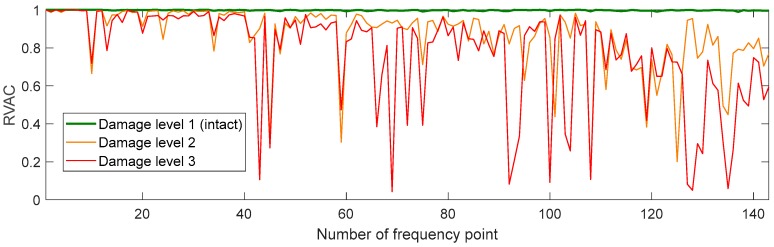
RVAC curves in three damage levels.

**Figure 7:**
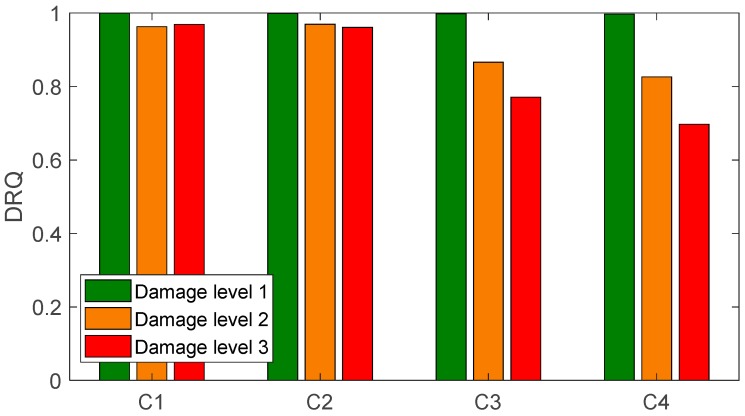
DRQ values of the three damage levels for different frequency ranges.

We found a total of 216 peaks in the singular value spectrum of the measurement 1 in the range of 0 to 100 kHz. Figure 4 gives an overview of this singular value spectrum. As examples, the peaks in frequency ranges from 0 to 4000 Hz, and 78 kHz to 82 kHz are shown in more detail with their peak number. The ODSs (here as the velocity data) at the corresponding frequency points are used to calculate dominate (sub-) ODSs by Equation (4), i.e., $u_{1}\left(\omega \right)$, with respect to noise robustness for the calculated RVAC values. We found that the velocities measured in laser beam directions, i.e., the raw velocities, are more reliable than the transformed 3D vibrations for damage detection. Therefore, we calculate RVAC values based directly on raw velocities. This will be discussed in Section 4. Figure 5 shows the RVAC values between the first measurement and the other four measurements. The corresponding DRQ values are shown in Table 2. All RVAC curves are strongly correlated with each other. The DRQ values are all over 0.98. The RVAC values at several frequency points are clearly lower than the rest, for example at frequency point number 121 (Figure 5). This may indicate that the RVAC values at these frequency points are very sensitive to operational conditions and the setup of the experiment. Therefore, we unselected the frequency points, at which at least one of the four RVACs is smaller than the mean value of the four DRQ values (0.9900) in Table 2, for example the frequency point 78. At this frequency point, only the RVAC value between measurements 1 and 2 (blue line) is lower than 0.9900; subsequently, there are in total 143 frequency points. The damage detection is then based on ODSs at these frequency points. Figure 6 shows the RVAC values in three different damage levels at the selected 143 frequency points. Table 3 gives a short overview of the selected resonance frequencies in three different frequency ranges. The green line is the reference RVAC curve (damage level 1). It is calculated by taking the mean values from the four RVAC curves in Figure 5 at each frequency point. The RVAC curve for damage level 2 in yellow is calculated from measurement 1 and measurement 6, the latter of which is measured on the cantilever beam in damage level 2. The red RVAC curve is calculated from measurement 1 and measurement 7, the latter of which is measured on the cantilever beam in damage level 3. The RVAC values in damage level 1 (the green line) are much higher than the rest. The difference between damage level 2 and damage level 3 becomes clearer in the frequency range over the frequency point 41, which is about 17.100 kHz. In order to take an overview of this, DRQ values in four different frequency ranges for the RVAC curves are shown in Figure 6. These four cases are named C1 for 0–1500 Hz, C2 for 0–17 kHz, C3 for 0–100 kHz and C4 for 17–100 kHz. The results are shown in Figure 7. The DRQ value with selected resonance frequency points up to 17 kHz (case C1 and C2) cannot identify the difference among the three damage levels clearly. With all selected frequency points in the entire measurement frequency range (case C3) or only above 17 kHz (case C4), the difference is much more clear. This is the advantage of measuring in a wide frequency range. Therefore, it can be concluded that, with our measurement methodology, a local damage due to a blind hole on a cantilever beam can be detected under operational conditions of a rotating machine. The DRQ value gives a single value damage index as an indication of damage existence and strength.

## 4. Discussion

As mentioned in Section 3, the 3D velocities estimated from the raw velocities are subject to measurement uncertainty. Errors in the velocity measurement could be further amplified for the velocities in Cartesian coordinates due to the inversion by the coordinate transformation if the condition of the transformation matrix is not good enough [38]. Several authors attempted to find optimal Euler angles to reduce this effect [39,40], but it is difficult to keep all the Euler angles optimal in practice, especially if vibration is measured with a lot of channels. The raw measurement data are therefore used as ODS (i.e., h(t)) to calculate the RVAC values because they are not influenced by the measurements of Euler angles and are more reliable.

**Figure 8:**
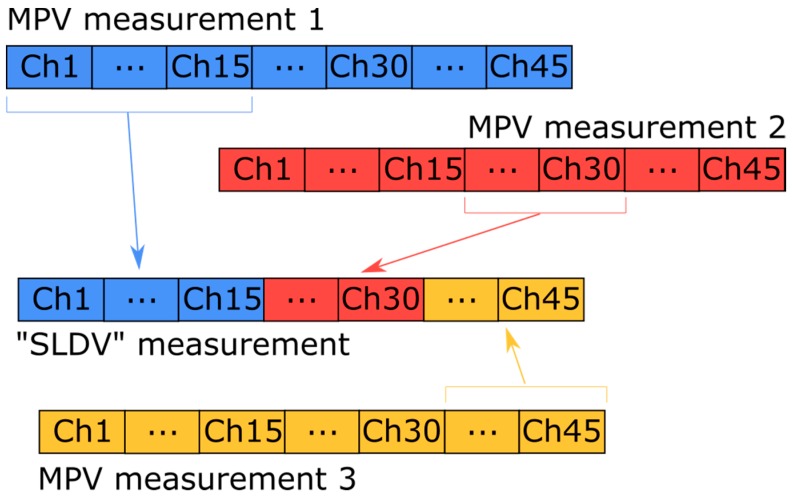
Schematic representation of the built “SLDV” measurement.

**Figure 9:**
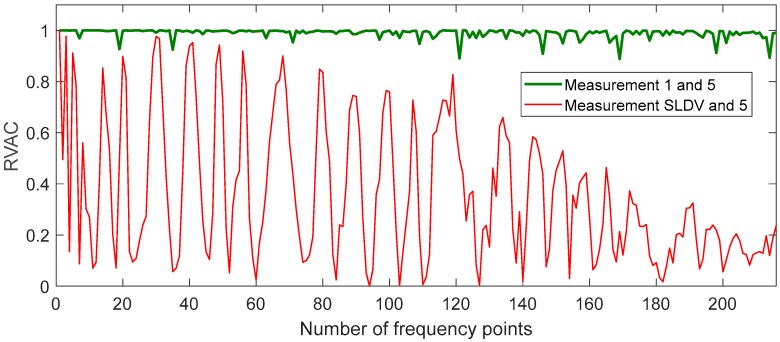
RVAC curves comparison between MPV measurement and SLDV measurement.

**Figure 10:**
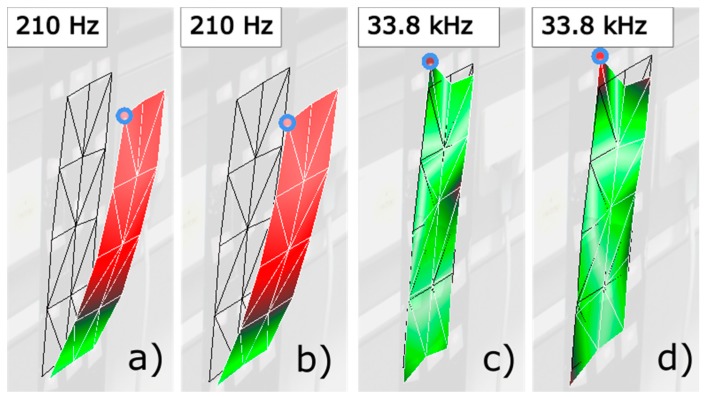
ODSs comparison from the cantilever beam in damage levels 2 and 3 at two frequencies with measurement points (circled in blue) as an example.

Before capturing the measurements on the cantilever in damage levels 2 and 3, we acquired five measurements on the cantilever in its intact state to obtain reliable reference RVAC and DRQ values. In addition, we used the measurements 1, 2, 3, and 5 to illustrate that simultaneous measurement is more reliable for damage detection. We built a SLDV “measurement” from the first three measurements. This means that the first 15 channels in the SLDV measurement are the same as the first 15 channels in the first MPV measurement; the second 15 channels in SLDV measurement are the same as the corresponding channels in the second MPV measurement and so on (see Figure 8 for illustration of the SLDV “measurement” routine). Then, the RVAC values between the MPV measurement 5 and the SLDV “measurement” are calculated. The result is shown in Figure 9. The RVAC curve between measurements 1 and 5, as a reference, is the green line, which was also shown in Figure 5 earlier in this paper. The RVAC curve of SLDV (red line) is extremely distorted and the DRQ value is only 0.3628. The low RVAC values in this case are results from the random transient friction between the electric motor and the cantilever beam during the measurement. Consequently, the synchronizing between friction excitation and measurement becomes practically impossible, and leads to a loss of the phase relation for the scanning. Furthermore, the amplitudes of the friction excitations during different measurements are not same, which falsify the ODSs from the “SLDV” measurement. Therefore, scanning measurement techniques, such as SLDV, may result in low RVAC values, even if a machine operates under practically constant rotational speed. These problems do not exist for MPV; however, the measurement grid has a maximum of 48 1D points. The number of measurement points is much lower than other full-field techniques like digital image correlation [41], but the detection frequency range is much wider. In the high frequency range, a dense measurement grid is required to measure the ODS completely because the structure-borne sound wavelength may be shorter than the distance between two adjacent measurement points [42]. Therefore, ODSs measured by MPV in frequency ranges over 20 kHz may be not complete, but the correlation between the measured ODSs from two different measurements on the same structure should still be equal to one. The RVAC values give the correlation quantitatively and are therefore sensitive to small changes in response signals. Figure 10 gives an example of two ODS pairs at two different resonance frequency points. The point circled in blue on each ODS is the first measurement point, for example. The ODSs (a) and (b) are measured on the cantilever in damage levels 2 and 3 at 210 Hz, respectively. Both ODSs are completely measured. The RVAC value for this ODS pair is 0.9999. The ODSs (c) and (d) are also from damage levels 2 and 3 but at about 33.8 kHz. They seem to be not completely measured. There is a small difference between them, which can be found by the corresponding RVAC value (0.6574) much more clearly. The ODSs at 33.8 kHz are not excited by the machine operation, but by the Nd:YAG laser excitation. The difference between the two ODSs at 33.8 kHz helps us to detect the structural damage earlier. Overall, our methodology represents a compromise of all simultaneous measurement techniques.

LDV and MPV also have limitations, which have to be considered. Acceleration sensors exist with a better resolution as it is achieved by the laser interferometric technique. As an example, we compared the sensitivity of our MPV system to a high-frequency acceleration sensor Type 8309 (Brüel and Kjær A/S, Nærum, Denmark). The resolution is expressed as root mean square (RMS) background noise acceleration in the specification of the acceleration sensor Type 8309. In order to make a fair comparison, we estimated the same value of MPV-800 approximately for the same bandwidth as the acceleration sensor Type 8309. The noise-limited resolution of MPV-800 at 50 kHz $\sqrt{S_{v}\left(f=50\;\mathrm{kHz}\right)}$ is $0.13\;\frac{\mu m}{s\sqrt{\mathrm{Hz}}}$ as per its technical specifications (frequency range: 0–100 kHz, main cable length: 10 m). $S_{v}\left(f\right)$ is the squared velocity spectral density of background noise at frequency *f*; $S_{a}\left(f\right)$ and $S_{u}\left(f\right)$ are the accelerration and displacement form of it. The noise limited resolution is defined as the signal amplitude (RMS) at which the SNR is 0 dB and with 1 Hz spectral resolution. Its displacement form at 50 kHz is then $\sqrt{S_{u}\left(f=50\;\mathrm{kHz}\right)}\;=\;\sqrt{S_{v}\left(f=50\;\mathrm{kHz}\right)}/\left(2\pi \right)/\left(50\;\mathrm{kHz}\right)\approx 0.4138\;\frac{\mathrm{pm}}{\sqrt{\mathrm{Hz}}}$. Assuming that the displacement noise spectrum is flat (white noise from electronics), i.e., $\sqrt{S_{u}\left(f\right)}=\mathrm{const}.=\;\sqrt{S_{u}\left(f=50\;\mathrm{kHz}\right)}$, the RMS background noise acceleration of MPV-800 $a_{n,\mathrm{MPV}}$ can be estimated by

(6) $$\begin{array}{ccc} a_{n,\mathrm{MPV}} & = & {\left(\int_{f_{\min}}^{f_{\max}}S_{a}\left(f\right)\;df\right)}^{\frac{1}{2}}\\ & = & {\left(\int_{f_{\min}}^{f_{\max}}{\left(2\pi f\right)}^{2}{\left(2\pi f\right)}^{2}S_{u}\left(f\right)\;df\right)}^{\frac{1}{2}}\\ & = & {\left(16\pi^{4}{\left(0.4138\;\frac{\mathrm{pm}}{\sqrt{\mathrm{Hz}}}\right)}^{2}\int_{f_{\min}}^{f_{\max}}f^{4}\;df\right)}^{\frac{1}{2}} \end{array}$$

according to Parseval’s theorem [43]. $f_{\min}$ and $f_{\max}$ are the lower and upper limit of the bandwidth. The acceleration sensor Type 8309 has an RMS background noise acceleration of $230\;\frac{\mathrm{mm}}{s^{2}}$ in the frequency range from 1 Hz to 54 kHz according to its technical specifications. In the same frequency range, the RMS background noise acceleration of MPV-800 is about $4950\;\frac{\mathrm{mm}}{s^{2}}$ as per Equation (6). Increasing the power of the laser excitation may overcome this limitation. Furthermore, the measurement object has to provide optical access for optical measurement techniques. Surface preparation on the measurement object may be necessary to achieve optimal signal conditions. Moving measurement objects, such as a rotating propeller, need a relevant tracking system for the vibration measurement. In cases where these limitations are avoidable, the demonstrated method is a reliable choice to detect damage on working machinery or test benches.

## 5. Conclusions

The experiments on the cantilever beam in three damage levels confirm that our measurement methodology is capable of detecting local damage on a cantilever beam under operational influences due to the rotating machine. The DRQ gives a quick indication for the damage existence. If the DRQ drops continuously over the operation time, the damage grows. The machine operation should be stopped if the damage level reaches a critical state regarding safe machine operation. Further examinations will then be carried out under laboratory conditions by full-field techniques like SLDV. With the measurements from SLDV, the damage will be localized by high spatial resolution-based methods such as modal curvature [44,45] or local defect resonance [16].

The RVAC value used here is a common damage index for damage detection. Robust damage index in post-processing is still a research topic today. Improved damage index will increase the damage detection efficiency of our measurement setup. The laser excitation methods will be further investigated to increase SNR of vibration signals. Better solutions to position the measurement heads of MPV can be carried out to make more reliable ODS measurements.

## Figures and Tables

**Table 1:** List of damage levels.

	Damage Level 1	Damage Level 2	Damage Level 3
Diameter of the blind hole in mm	0	4.30	4.30
Depth in mm	0	2.66	4.09

**Table 2:** DRQ values from the four measurement pairs in Figure 5.

Measurements 1 and 2	Measurements 1 and 3	Measurements 1 and 4	Measurements 1 and 5
0.9904	0.9896	0.9905	0.9894

**Table 3:** An overview of the selected resonance frequency points.

**Frequency Range in kHz**	0–35	35–70	70–100
**Number of Resonance Frequency Point**	1–70	71–120	121–143
